# Improving compliance with placental histopathology through labour ward storage and clear referral criteria: a two-site quality improvement report

**DOI:** 10.1136/bmjoq-2026-004321

**Published:** 2026-08-26

**Authors:** Lisa Story, Anangsha Kumar, Dee Laker, Spyros Bakalis, Colette McGilchrist, Daniel Cromb, Andreas Marnerides, Rebecca Smith, Ashleigh McDonald, Paul Cawley

**Affiliations:** 1 Women’s Services, Guy's and St Thomas’ NHS Foundation Trust, London, England, UK; 2 Department of Women and Children’s Health, King’s, London, England, UK; 3 Department of Early Life Imaging, King’s College London, London, UK; 4 Department of Cellular Pathology, Guy's and St Thomas’ NHS Foundation, London, UK; 5 Royal Berkshire Hospital, Reading, England, UK; 6 Department of Neonatology, Evelina London Children’s Hospital, Guy's and St Thomas’ NHS Foundation Trust, London, UK

**Keywords:** Obstetrics and gynecology, Pathology, Quality improvement

## Abstract

Placental histopathology is central to good perinatal care, offering insight into antepartum and intrapartum events, informing neonatal management and supporting counselling for future pregnancies. Despite clear national guidance, many placentas that meet criteria are not sent for evaluation. Reasons are multifactorial, including inconsistent awareness of indications and unclear post-delivery processes. Two UK maternity units, St Thomas’ Hospital in London and the Royal Berkshire Hospital in Reading, identified local variations in practice that led to placentas not being appropriately sent. At St Thomas’ Hospital, a baseline audit performed as part of a preterm birth research study revealed only 80% of eligible deliveries had placental examination. At the Royal Berkshire Hospital, a coroner’s inquest after an unexpected neonatal death, highlighted the absence of indicated placental histology, prompting a recommendation for 48-hour retention of all placentas. A quality improvement project using Plan Do Study Act cycles was implemented at both sites. Interventions included installation of dedicated labour ward fridges for 48-hour storage with temperature monitoring, targeted education on indications for placental histology and daily cross-checking of neonatal admissions to ensure eligible placentas were sent. The primary outcome was the proportion of placentas meeting criteria sent for histopathology, focusing on deliveries <32 weeks and intrauterine deaths >18 weeks. National investigation reports at St Thomas’ Hospital were also reviewed for missing histology. Labour ward storage proved feasible and effective. At the Royal Berkshire Hospital, submission rates improved from 88% to 97% for intrauterine deaths and from 71% to 100% for very preterm births. At St Thomas’ Hospital, preterm submissions increased from 80% to 96% after education and 98% after storage, with intrauterine death remaining at 100%. Missing histology in national investigations reduced from 12% to 0%. Short-term placental storage is a low-cost, scalable intervention aligned with national maternity safety priorities.

**WHAT IS ALREADY KNOWN ON THIS TOPIC**

- Many placentas are not sent for histopathological assessment despite the existence of clear national and international guidelines. This is likely due to inconsistent awareness of referral criteria and lack of post-delivery storage pathways.

**WHAT THIS STUDY ADDS**

- We have demonstrated that short-term placental storage, education regarding the Royal College of Pathologists’ criteria for referral and cross-checking of neonatal admissions to ensure placental histology had been sent where indicated improved the percentage of placentas appropriately referred for histopathological assessment in two NHS maternity units.

**HOW THIS STUDY MIGHT AFFECT RESEARCH, PRACTICE OR POLICY**

- We have shown that 48-hour labour ward placental storage, with cross-checking of neonatal admissions and increased education, is a simple solution that could inform national recommendations on placental retention, improving perinatal pathology access and ultimately improving patient care.

## Problem

 St Thomas’ Hospital (London) and the Royal Berkshire Hospital (RBH, Reading) are two large maternity units in South England. St Thomas’ Hospital is a tertiary referral centre and delivers over 6000 babies, and the RBH is a district general unit with approximately 4000 deliveries a year. Despite well-established Royal College of Pathologists’ (RCPath) guidance and local Trust guidelines detailing the indications for histopathological assessment of the placenta, both units noted this was done inconsistently.

At St Thomas’ Hospital, a baseline audit for a research study, focusing on very preterm birth <32 weeks gestation, found that only 80% of very preterm deliveries had placental histology performed, when compliance should have been 100%. This was thought to be attributable to uncertainty about indications for referral from delivery suite staff. Introduction of placental storage was thought to provide an additional mechanism as a safety net to any cases missed at the time of delivery.

At the RBH, a serious clinical incident led to a coroner’s inquest that identified the absence of placental histology despite clear indications. The coroner recommended that all placentas should be stored for at least 48 hours after delivery to avoid future omissions. This external mandate prompted a change in clinical pathways to ensure its implementation.

For both hospitals, addressing limited refrigeration capacity on the labour ward and education regarding the indications and importance of referral for histopathological evaluation were deemed crucial. Both units sought to implement a sustainable strategy to improve adherence to the guidelines for histopathological evaluation of placentas, reduce missed diagnostic opportunities but also create a system by which placentas could also be sent if a baby was well at birth but deteriorated in the first 48 hours after delivery. This work aimed to increase the proportion of eligible placentas submitted for histopathology by introducing standardised 48-hour labour ward storage, cross-checking neonatal admissions and education regarding appropriate referral criteria.

## Background

The placenta plays a critical role in facilitating nutrient and gaseous exchange between the maternal and fetal circulations.^1^ Its morphology and function continually develop and adjust during the course of a pregnancy and where these processes occur suboptimally a number of obstetric conditions can result.

Pathologic assessment of the placenta can offer clinical value in such situations. Proper examination can afford insight into antepartum and intrapartum events that cannot only explain outcomes of that pregnancy but provide prognostic information for the health of the child and mother and help direct future pregnancy management.^2^ It can also play a critical role in serious incident reviews, audits of patient care and medicolegal cases.^3^

Currently, RCPath UK recommends that placentas be routinely sent for the reasons listed in table 1.^3^

However, despite the fact that placental histology is extremely valuable and guidelines are clear as to which placentas should be sent for analysis, studies have indicated that between 25% and 65% of placentas were not examined.^4^ A modified Delphi consensus of 16 placental pathologists published in the *American Journal of Obstetrics and Gynaecology* in 2023 recommended retaining placentas that do not meet the criteria for pathological assessment at delivery for at least 72 hours where feasible.^5^ The basis of this recommendation was that delayed perinatal morbidity and/or subsequent early neonatal mortality might occur when the indication for placental histology is not apparent at the time of delivery. Obtaining this information in these circumstances was suggested to impact prognosis and recurrence risk and reduce medicolegal action.^5^ This has been implemented in some hospitals in both the USA and UK for many years, and storage can be as long as 10 days^5^; however, the practice is less widespread in the UK.

**Table 1:** Royal College of Pathologists UK’s criteria for histopathological examination of placenta

1.	All stillbirths (antepartum or intrapartum)
2.	Miscarriages (14–23+6 weeks)
3.	Severe fetal distress (definition: pH <7.05 or base excess ≤12 or scalp lactate >4.8 mmol/L)
4.	Preterm birth (<32+0 weeks)
5.	Infants with fetal growth restriction (defined as birth weight <3^rd^ centile or a drop in fetal growth velocity of >2 quartiles or scalp lactate >4.8 mmol/L)
6.	Abnormal umbilical artery Dopplers (absent or reversed end diastolic flow)
7.	Fetal hydrops
8.	Early-onset (<32 weeks gestation) severe pre-eclampsia requiring iatrogenic delivery
9.	C-section peripartum hysterectomy for morbidly adherent placenta
10.	Severe maternal sepsis requiring intensive care admission
11.	Fetal sepsis requiring ventilation or level 3 NICU admission
12.	Massive placental abruption with retroplacental clot
13.	Monochorionic twins with twin-to-twin transfusion syndrome

NICU, neonatal intensive care unit.

## Measurement

To understand baseline performance, three metrics were selected. These were deemed to align with both national guidance as well as local priorities of the two units.

### Proportion of placentas from deliveries <32 weeks of gestation sent for histopathology

RCPath guidance suggests that all placentas should be sent for histopathological evaluation for very preterm delivery. Pathological findings are often noted, including chorioamnionitis. Both units collected data from their electronic maternity records for a 6-month baseline period (St Thomas’ Hospital, January–June 2023; Royal Berkshire, August 2022-January 2023), including all live births <32 weeks and recording if placental histopathological evaluation occurred.

### Proportion of placentas submitted following intrauterine deaths ≥18 weeks

Placental histology from all intrauterine deaths (IUFDs) after 18 weeks of gestation is also recommended. This is the gestational age at which these two units transition from gynaecology to obstetric services. The two units therefore reviewed all such cases during the same baseline period used for very preterm deliveries to reassess rates of appropriate histopathological assessment.

### Proportion of maternity and neonatal safety investigation/healthcare safety investigation branch investigation reports noting missing placental histology

Healthcare safety investigation branch investigation (HSIB; 2017–2023) and later maternity and neonatal safety investigation (MNSI; 2022 onwards) reports are independent, national level safety investigations for intrapartum deaths, early neonatal deaths, neonatal hypoxic ischaemic brain injury at term and maternal deaths in the UK. Placental histology often forms a crucial part of these investigations. As part of this work, all reports were reviewed from patients who delivered at St Thomas’ Hospital between 2017 and the date of implementation, specifically assessing the absence of placental histology.

In addition, feedback from pathology staff, neonatal clinicians and midwives was informally gathered to understand facilitators and barriers to implementation.

NHS Research Ethics Committee approval was not required because the project was classified locally as quality improvement/service evaluation.

## Design

The project team at both sites consisted of obstetricians, neonatologists, midwives, pathologists and risk leads/quality improvement staff. A Plan Do Study Act framework was developed:

### Plan

The aim for both sites was to increase the proportion of placentas submitted for histopathology as per guidelines, specifically focusing on IUFDs >18 weeks, preterm births <32 weeks and cases where HSIB/MNSI reports had been generated. It was hypothesised that implementing a standardised 48-hour storage system, cross-checking neonatal admissions and increasing staff awareness would improve compliance.

### Do

The main intervention was the purchase and installation of a dedicated placenta refrigerator on both of labour wards. Education was also rolled out during this period regarding indications for placental storage and processes of keeping the placentas on labour ward, for example, labelling requirements. This occurred through handovers, emails, posters and training sessions. Neonatal admissions were reviewed daily to identify cases where placental pathology may have been indicated but not yet submitted to ensure no placentas were missed.

### Study

Data on the number of placentas sent for histology were reviewed, and informal feedback was collected from staff to identify barriers, including inadequate labelling, equipment challenges or workload-related issues. When the new processes were introduced, some refinements were required (eg, adopting double bagging after initial leakage issues).

### Act

Both units implemented routine 48-hour storage as standard practice. Guidelines were updated, and education programmes with regard to indications for placental histology and the processes of storing the placentas after delivery were implemented .

Cross-checking of neonatal unit admissions was also instigated.

Patients were not actively involved in the design or dissemination of this work.

## Strategy

Teams at both sites included obstetricians, neonatologists, pathologists and midwives. This work was registered as quality improvement projects/audits at Guy’s and St Thomas’ NHS Foundation Trust and at RBH (numbers 17 580 and N5379 respectively).

### St Thomas’ Hospital

The initial phase at St Thomas’ Hospital focused on raising staff awareness of the situations that would require placentas to be sent for pathological assessment. Posters summarising RCPath indications were displayed on the labour ward, email reminders were sent and the issue was raised at maternity handovers. This educational drive did improve compliance: the proportion of placentas sent from very preterm births (<32 weeks) increased from 80% to 96%. Despite this, education alone could not address situations in which indications emerged only after delivery, for example, if a baby became unexpectedly unwell on the postnatal ward. Without an available placenta stored on site, these cases would still be missed.

To address this limitation, St Thomas’ trialled an initial storage system in which all placentas were retained for 7 days, in accordance with practices used in several international centres. However, this approach proved impractical as placentas began to deteriorate within the refrigerator, generating odour and making handling unpleasant for staff, the ward lacked the capacity to safely accommodate a full-week volume, and infection control concerns were also raised. Following a feasibility review, the team refined the retention period to 48 hours, this was deemed long enough to capture neonatal deterioration or evolving clinical concerns, yet short enough to remain manageable within labour ward space, infection control standards and staff workload. A mortuary grade fridge was subsequently ordered, with separate shelves allocated by day to make the storage process as simple as possible. The top shelf was reserved for placentas identified as needing transfer to histopathology at the time of delivery.

Early in the process, further challenges also became apparent. Placentas stored in single bags frequently leaked, contaminating the fridge, creating hygiene issues and making handling unpleasant for staff. As a result, a double bagging system was introduced using two clinical waste bags. This reduced leakage and resulted in fewer issues for staff using the fridge. Daily cross-checking of neonatal admissions was also undertaken to ensure that no placentas had been missed from neonatal admissions where they were required and that any neonates who had been admitted to the unit after delivery could also be captured. This safety net proved valuable in identifying occasional cases in which placentas had been stored but not sent. This allowed the team to identify process failures, particularly around labelling and referral, and informed targeted re-education.

Education continued during this time with further email communications and posters disseminated by the risk team for display on the labour ward and by the placenta fridge. These processes helped embed the new pathway within routine clinical practice.

### Royal Berkshire Hospital

At the RBH, the decision to introduce 48-hour placental storage was driven by a coroner’s directive following an adverse perinatal event. The Trust responded by also implementing a labour ward storage system for all placentas. The initial plan had been to use a standard clinical refrigerator, but this soon proved impractical because of the structural strength required to accommodate the weight and volume of stored placentas, and it did not provide continuous temperature monitoring capability, which is essential for specimen integrity.

The unit therefore purchased a mortuary-grade refrigerator, though space limitations meant that shelf capacity remained modest (it was not possible to have a separate shelf for each day of the week). To maximise the space that was available and provide some organisation, placentas were bagged, labelled and placed in grouped batches within designated buckets inside the fridge. A specific labelling system was introduced, requiring each bag to carry a sticker with the patient’s name, date of birth, hospital number and date of delivery.

The RBH also created a clear pathway for women wishing to take their placenta home. Midwives first obtained verbal consent for temporary storage, explaining that retention for 48 hours was recommended to ensure that placental examination remained an option should the baby become unwell (no woman declined storage during the evaluation period). Women wishing to take their placenta home immediately after delivery were supported to do so as long as they were aware why retention was recommended. The other option offered was that they could return to collect their placentas after the initial 48-hour storage period whereby it was stored in a designated biobin. A patient information leaflet was also created to explain the rationale for the 48-hour storage and outline all of the available choices to women. This ensured that the storage pathway remained compatible with patient choice while preserving the option of clinically indicated histopathological examination if concerns emerged after delivery.

Similar to St Thomas’ Hospital, education accompanied the rollout of the new system of the 48-hour placental storage. The Trust updated its local guidelines, and briefings were provided during handovers to ensure consistent understanding across the multidisciplinary team. Posters were also used on labour ward and on the placental fridge itself to highlight the new processes. As at St Thomas, no additional staff were required to implement this change, and all processes were absorbed into existing workflows.

## Results

The intervention periods were August 2023 at St Thomas’ Hospital and February 2023 at the RBH. Evaluation following the change in process (48-hour placental storage) occurred from February to June 2024 at St Thomas’ Hospital and from March to August 2023 at the RBH.

### Primary outcomes

**Figure 1:**
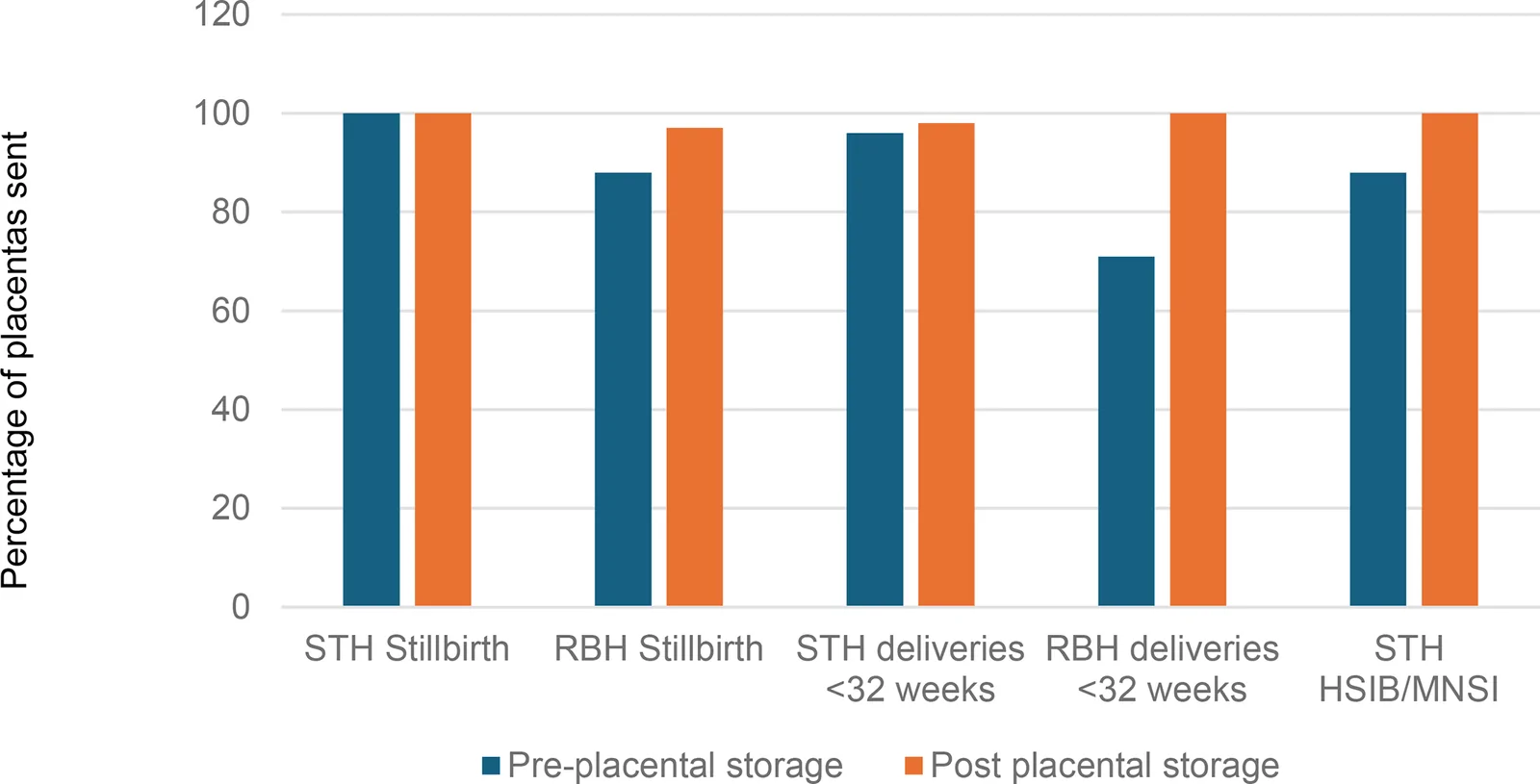
Bar chart indicating the percentage of placentas sent for histology pre-implementation and post-implementation at St Thomas’ Hospital (STH) and Royal Berkshire Hospital (RBH).

Results for compliance for the percentage of placentas correctly sent for histopathological assessment can be seen in table 2 and figure 1. The intrauterine death histopathology submission rate and the MNSI/HSIB missing-histology indicator were treated as separate measures.

**Table 2:** Placental histopathology submission rates for stillbirth and deliveries <32 weeks before and after implementation of placental storage at Royal Berkshire Hospitaland St Thomas’ Hospital and the number of HSIB/MNSI reports noting missing placental histology before and after the implementation of the Royal College of Pathologists UK’s criteria for histopathological examination of placenta

Hospital	Indicator	Pre-intervention	Post-intervention
Royal Berkshire Hospital	Stillbirth (IUFD ≥18 weeks)	30/34 (88%)	29/30 (97%)
Royal Berkshire Hospital	Deliveries <32 weeks	10/14 (71%)	21/21 (100%)
St Thomas’ Hospital	Stillbirth (IUFD ≥18 weeks)	10/10 (100%)	19/19 (100%)
St Thomas’ Hospital	Deliveries <32 weeks	49/51 (96%)	51/52 (98%)
St Thomas’ Hospital	HSIB/MNSI reports noting missing placental histology	3/26 (12%)*	0/7 (0%)†

* One maternal death at home during postpartum was excluded, as placental histology would not have been expected after discharge.

† One referral occurred beyond the 48-hour storage window and was not included.

HSIB, Healthcare safety investigation branch; IUFD, intrauterine fetal death; MNSI, maternity and neonatal safety investigation .

In addition, during the evaluation period after the intervention, no temperature-related losses occurred, labelling improved, and the operational burden was absorbed within the existing staffing structure. Some placentas at St Thomas’ Hospital could not be identified as had not been labelled, but this prompted targeted re-education.

Education improved early compliance but did not solve the issue in which a baby became unwell after the immediate delivery period (when previously the placenta would automatically have been discarded). The 48-hour storage window addressed this and, together with cross-checks and prompts, produced near-complete compliance in high-risk cohorts, including 100% submission for <32 week births at RBH.

## Lessons and limitations

This project demonstrates that the 48-hour storage of all placentas on the labour ward is both feasible and practical within two busy UK maternity units: a tertiary referral unit and a district general. The intervention, purchase of a designated fridge, education and cross-checking neonatal admissions, required minimal financial investment, and additional workloads were manageable within existing staffing resources.

Several important lessons were highlighted from this work. First, although education alone improved compliance, it could not address the challenges posed by clinical scenarios in which indications for placental histology only became apparent after birth, for example, when a baby collapsed on the postnatal ward. Improving compliance therefore also required an additional intervention, that is, storage of all placentas on the labour ward for 48 hours. This provided a safety net if individual staff erroneously did not send the placenta for histological analysis at the time of delivery as neonatal admissions were cross-checked with placental histology requests as well as cases of unexpected deterioration of a neonate in the first 48 hours. Second, operational details affected the implementation, but solutions were found. Examples included double bagging placentas at one unit to reduce leakage and improve hygiene. The importance of accurate labelling was also highlighted during this work. Cleaning schedules and checking temperature logs and mechanisms to remove placentas after 48 hours were also required. Together, these practical refinements increased staff confidence and improved the workflow.

The project had a few limitations. First, the two sites introduced the storage system for different reasons and at different times. This led to different baseline period data, reducing direct comparability, and different assessment times in relation to the intervention. Second, the sample sizes were small, and no data were available for HSIB/MNSI reports from the RBH. Neonatal deterioration may also have occurred outside of the 48-hour window, but this timeframe provided a pragmatic approach as to what was achievable within two busy maternity units. Although other authors have suggested placental retention for at least 72 hours where feasible, our project did not directly compare 48-hour and 72-hour storage. At St Thomas’ Hospital, longer retention was initially trialled but was not operationally sustainable because of storage capacity, odour, specimen deterioration and infection-control concerns. The pathway was therefore refined to 48 hours as a pragmatic balance between capturing early neonatal deterioration and maintaining a feasible labour ward process. Future work should evaluate whether the 72-hour retention period provides additional benefit over 48-hour retention. This preliminary work only focused on compliance for high-risk groups with clear national referral criteria and reliable data capture such as very preterm births and stillbirths, but more situations warrant histopathological assessment of the placenta. These indicators were therefore pragmatic and chosen based on clinical importance. Future work should evaluate compliance across the full range of RCPath referral criteria. Unlabelled placentas were also highlighted as a risk area, and this could potentially be overcome by using digital barcoding in the future.

The context preceding the intervention may also have introduced bias. At the RBH, the coroner’s inquest created heightened organisational awareness of missed placental histology, whereas at St Thomas’ Hospital, the baseline audit increased sensitivity to compliance with referral criteria. These factors may have contributed to improvements alongside the formal intervention. However, this also reflects the real-world context of quality improvement work, where interventions are commonly triggered by identified safety gaps.

As this was a quality improvement project using before-and-after comparisons, causality cannot be definitively attributed to the organisational changes implemented. The observed improvements in placental histopathology submission rates occurred after the introduction of education, labour ward storage and neonatal admission cross-checking, but the design does not exclude the influence of other contemporaneous factors, including increased staff awareness following the baseline audit and coroner’s inquest. The findings should therefore be interpreted as evidence of association and feasibility rather than definitive proof of causation.

Sustainability is an important factor moving forward and was considered during implementation, but formal long-term sustainability assessment was not undertaken within the evaluation period. Staff turnover can be high among resident doctors and more junior staff, which means that education programmes need to be ongoing. Incorporating education into mandatory training sessions in maternity such as Practical Obstetric Multi-Professional Training (PROMPT) can be explored in the future. Both units plan to continue monitoring compliance with placental histology criteria to ensure the system remains robust over time as it is continuously being refined. Finally, the development of wider national guidance, supported by labour ward posters, handover reminders and ongoing staff education could facilitate broader adoption. Future sustainability measures may include digital barcoding or electronic prompts to reduce labelling errors and improve traceability. An important point highlighted from this work is that if a coroner raises concerns about processes in one maternity unit and mandates a change in practice, this learning point should then be disseminated to all maternity units to improve standards nationally.

## Conclusion

This project demonstrates that introducing a 48-hour placental storage system on the labour ward, alongside education and cross-checking, was feasible and was associated with improved compliance within two busy NHS maternity units. Although the rationale behind which placental storage was instigated in each unit differed, the outcomes converged with both hospitals improving the percentage of placentas that were appropriately sent for histopathological evaluation in accordance with national RCPath criteria. Placental pathology remains fundamental to understanding adverse perinatal events, guiding neonatal management, informing counselling for future pregnancies as well as strengthening the quality and credibility of maternity investigations.

Education, 48-hour placental storage on labour ward and cross-checking placentas sent for histology with neonatal admissions require minimal infrastructure and can be incorporated into existing workflows and staffing structures. Its implementation in two separate units highlights its potential for wider national adoption, particularly in the context of ongoing efforts to enhance perinatal review processes and promote system wide learning from serious incidents. However, longer-term monitoring is required to assess sustainability. Most importantly, when a coroner identifies a safety critical process change for one maternity unit, this should be highlighted to all maternity units nationally to strengthen collective maternity safety.

Future work should focus on evaluating wider implementation in all areas of the UK and ensuring the system is sustainable in individual units. Digital solutions such as barcoding placentas can be explored as well as approaches to improve processes, for example, vacuum sealing placentas on labour ward, which may improve hygiene and ease of handling. Learning from the experience of individual maternity units may provide a pathway toward universal adoption, ensuring all families benefit from the clinical value of high quality placental examination.

## Data Availability

No data are available.
